# Blinding Trachoma in Postconflict Southern Sudan

**DOI:** 10.1371/journal.pmed.0030478

**Published:** 2006-12-19

**Authors:** Jeremiah Ngondi, Francis Ole-Sempele, Alice Onsarigo, Ibrahim Matende, Samson Baba, Mark Reacher, Fiona Matthews, Carol Brayne, Paul Emerson

**Affiliations:** 1 Department of Public Health and Primary Care, Institute of Public Health, University of Cambridge, Cambridge, United Kingdom; 2 Christian Mission Aid, Nairobi, Kenya; 3 The Carter Center, Nairobi, Kenya; 4 Ministry of Health, Government of Southern Sudan, Juba, Sudan; 5 Medical Research Council Biostatistics Unit, Institute of Public Health, Cambridge, United Kingdom; 6 The Carter Center, Atlanta, Georgia, United States of America; Bradford Royal Infirmary, United Kingdom

## Abstract

**Background:**

Trachoma is a leading cause of preventable blindness. Reports from eye surgery camps and anecdotal data indicated that blinding trachoma is a serious cause of visual impairment in Mankien payam (district) of southern Sudan. We conducted this study to estimate the prevalence of trachoma, estimate targets for interventions, and establish a baseline for monitoring and evaluation.

**Methods and Findings:**

A population-based cross-sectional survey was conducted in May 2005. A two-stage cluster random sampling with probability proportional to size was used to select the sample population. Participants were examined for trachoma by experienced graders using the World Health Organization simplified grading scheme. A total of 3,567 persons were examined (89.7% of those enumerated) of whom 2,017 were children aged less than 15 y and 1,550 were aged 15 y and above. Prevalence of signs of active trachoma in children aged 1–9 y was: trachomatous inflammation-follicular (TF) = 57.5% (95% confidence interval [CI], 54.5%–60.4%); trachomatous inflammation-intense (TI) = 39.8% (95% CI, 36.3%–43.5%); and TF and/or TI (active trachoma) = 63.3% (95% CI, 60.1%–66.4%). Prevalence of trachomatous trichiasis was 9.6% (95% CI, 8.4%–10.9%) in all ages, 2.3% (95% CI, 1.6%–3.2%) in children aged under 15 y, and 19.2% (95% CI, 17.0%–21.7%) in adults. Men were equally affected by trichiasis as women: odds ratio = 1.09 (95% CI, 0.81%–1.47%). It is estimated that there are up to 5,344 persons requiring trichiasis surgery in Mankien payam.

**Conclusions:**

Trachoma is a serious public health problem in Mankien, and the high prevalence of trichiasis in children underscores the severity of blinding trachoma. There is an urgent need to implement the surgery, antibiotics, facial cleanliness, and environmental change (SAFE) strategy for trachoma control in Mankien payam, and the end of the 21-y civil war affords an opportunity to do this.

## Abstract 2

A population-based, cross-sectional survey with two-stage cluster random sampling, conducted in Sudan's Mankien district, found a high prevalence of trachoma, with many patients in need of surgery for trachomatous trichiasis.

## Editors' Summary

**Background.:**

Trachoma is an infectious disease that it is cased by a bacterium, Chlamydia trachomatis. The infection can be passed from one person to another through contact with hands and clothes, and by flies. The disease develops gradually—while children are most susceptible to infection, they may not note its effects until adulthood, when scarring from repeated infections causes the eyelashes to turn inward (“trichiasis”). The cornea—the transparent front part of the eye—becomes damaged by the eyelashes and develops ulcers, and eventually blindness results. Trachoma is most common in poor rural communities living in overcrowded conditions with limited access to water and health care. As improved living conditions, better hygiene, and early treatment of the infection with antibiotics can prevent the disease—and surgery for trichiasis is very effective—trachoma is regarded as a preventable type of blindness. It is also described as a “neglected disease”; over seven million people have trichiasis and it causes an estimated 3.6% of the world's blindness, but it does not receive the attention that such a widespread and serious condition demands. The World Health Organization recommends a strategy for trachoma control known as SAFE—surgery, antibiotics, facial cleanliness, and environmental change.

**Why Was This Study Done?:**

The environment and living conditions in the southern Sudan are such that trachoma is likely to be common; there have been reports that this is the case, but reliable information has not been available. In part, this is because of the serious conflict in the country that lasted many years. In 2005 when the region became more peaceful, planning began in order to expand health programmes there. Good planning must be based on reliable information as to how many people are affected by different health conditions, including trachoma.

**What Did the Researchers Do and Find?:**

They focused on just one district of southern Sudan (Mankien), where they also conducted a survey that looked for all types of blindness. They selected 22 villages at random and sent people to conduct the survey who were experienced in recognizing trachoma and in using the WHO's system for grading the various stages of the disease, from early infection to blindness. Once in the village, the survey teams selected homes at random and examined the people there. A total of 3,567 people were examined, of whom 2,017 were children aged less than 15 years. The earliest stages of infection were very common indeed, particularly in children aged 1–9, over half of whom had some sign of infection. In adults, one in five had trichiasis caused by trachoma.

**What Do These Findings Mean?:**

The very high proportion of children with early stages of trachoma is of great concern and, based on the survey, over 5,000 people in Mankien are estimated to be in need of trichiasis surgery. The survey suggests that other parts of the southern Sudan are also likely to have similar problems. This is a particularly disturbing situation for a disease that should be very easy to prevent. Antibiotic treatment and preventive measures are also needed. The SAFE strategy must be urgently put into action. Further discussion of the implications of this study and of the methods the researchers used are found in two “Perspective” articles in this issue of *PLoS Medicine* (by Buchan and by Kuper and Gilbert).

**Additional Information.:**

Please access these Web sites via the online version of this summary at http://dx.doi.org/10.1371/journal.pmed.0030478.

- Basic information on trachoma may be found on Wikipedia, an internet encyclopaedia that anyone can edit
- More detailed information is available from the International Trachoma Initiative and Medline Plus
- The Carter Center implements trachoma control activities in Africa
- The SAFE strategy is described in detail in a document available on the Web site of the Carter Center

## Introduction

Trachoma is a neglected disease that affects those who live in the poorest conditions; it causes blindness, disability, stigmatization, and poverty. Caused by ocular infection with *Chlamydia trachomatis,* trachoma is the leading infectious cause of preventable blindness and is estimated to be responsible for at least 3.6% of all blindness worldwide [1]. The World Health Organization (WHO) estimates that active trachoma affects 84 million persons, and 7.6 million have trichiasis, the potentially blinding state of the disease, in 55 countries [2]. Blindness due to trachoma is preventable through the WHO-endorsed strategy of surgery, antibiotics, facial cleanliness and environmental change (SAFE) [3]. The WHO Alliance for the Global Elimination of Trachoma (GET 2020) has identified Sudan as one of the priority countries to be targeted for the implementation of the SAFE strategy.

Trachoma has long been known to be prevalent in parts of Sudan [4]. Recent surveys indicate that trachoma is a severe public health problem in the states of Eastern Equatoria and Upper Nile of southern Sudan [5]. Postconflict southern Sudan is characterized by poor or absent health infrastructure, widespread illiteracy, poor sanitation, lack of potable water, and extreme poverty, with over 90% of the population living off less than one dollar a day [6]. In Mankien payam (district), water is inaccessible most of the year, there are no latrines, people live in close proximity with cattle, eye-seeking flies are rampant, and hygiene practices are poor (The Carter Center, unpublished data). These factors have contributed to aggravating the trachoma situation. The prevalence of trachoma in Mankien is not known. However, during an eye surgery camp in November 2004, it was observed that trachomatous trichiasis (TT) was very common. Out of 368 patients who received eye surgery over a 2-wk period, 203 (55.2%) underwent trichiasis surgery (Christoffel-Blindenmission, unpublished data). As a response to this anecdotal evidence, we planned a cross-sectional population-based survey with the broad objective of estimating the prevalence of active trachoma and TT, and gauging the need for specific trachoma control interventions. The specific objectives were to: (1) estimate the prevalence of active trachoma signs in children aged 1–9 y; (2) estimate the prevalence of TT in persons aged 15 y and above; (3) estimate targets for trachoma control interventions; and (4) establish baselines for future monitoring and evaluation.

## Methods

### Study Site

A population-based cross-sectional study was conducted in Mankien payam, located in Unity state of southern Sudan in May 2005. Mankien has an estimated population of 50,000 persons [7]. This area has been severely affected by the 21-y conflict in southern Sudan due to its proximity to the oil fields. It comprises four bomas (subdistricts): Tam, Mandul, Kernyang, and Mankien. The main economic activities are cattle rearing and subsistence farming, and the Nuer are the predominant ethnic group.

### Sample Population

The sample size was calculated to allow for estimation of at least 50% prevalence of active trachoma (grades trachomatous inflammation-follicular [TF] and/or trachomatous inflammation-intense [TI]) in children aged 1–9 y within a precision of 10% given a 95% confidence limit and a design effect of 5. We also aimed to estimate at least 2.5% prevalence of TT in persons aged 15 y and above within a precision of 1.5% at 95% confidence limit and a design effect of 2 [8,9]. Therefore at least 480 children aged 1–9 y and 820 persons aged 15 y and above were to be examined.

A two-stage cluster random sampling design with probability proportional to size was used to select the sample. A cluster was defined as a village. Mankien payam has 73 villages in four bomas, one of which (Mankien boma, 12 villages) was inaccessible at the time of the survey due to insecurity and was therefore excluded from the sampling frame. The remaining 61 villages were eligible for selection: in the first stage, 22 villages were selected with probability proportional to the estimated population of the bomas. In the second stage, 25 households were selected from each village using the random walk method [10]. The first household was randomly selected by going to the middle of the village and spinning a pen after which the household nearest to the ball-tip of the pen was selected. Subsequent households were selected with the “next” household being defined as the one whose door was closest. All residents of selected households were enumerated and those present examined. An attempt was made to examine absentees by returning to households in which persons were absent on the day of the survey. Households in which all residents were not available were skipped. Due to logistical constraints, it was not possible to return to the village on a different day to follow up any absentees.

### Trachoma Grading

IECWs (integrated eye care workers) qualified in using the WHO simplified grading system were retrained by an experienced ophthalmic nurse (F. Ole-Sempele) [11]. This scheme comprises five stages: TF, TI, trachomatous scarring (TS), TT and corneal opacity (CO). Minimum accepted interobserver agreement was set at 80% and reliability assessed in two stages. In the first stage, trainee examiners identified trachoma grades using the WHO set of trachoma slides [8]. Those examiners who achieved at least 80% agreement then proceeded to the second stage of field evaluation. During field evaluation, a reliability study comprising 50 persons of varying age and sex were selected by the ophthalmic nurse to represent all trachoma grades. Each trainee examiner evaluated all 50 participants independently and recorded their findings on a preprinted form. Interobserver agreement was then calculated for each trainee using the ophthalmic nurses' observation as the “gold standard.” Only trainees achieving at least 80% interobserver agreement after the field evaluation were included as graders.

All persons living within each selected household who gave verbal consent were examined using a torch and a 2.5× magnifying binocular loupe. Each eye was examined first for in-turned lashes (TT), and the cornea was then inspected for COs. The upper conjunctiva was subsequently examined for inflammation (TF and TI) and scarring (TS). Both eyes were examined. Signs had to be clearly visible in accordance with the simplified grading system in order to be considered present. Alcohol-soaked cotton swabs were used to clean the examiner's fingers between examinations. Individuals with signs of active trachoma (TF and/or TI) were offered treatment with 1% tetracycline eye ointment. TT patients were referred to the health center where free surgery was available.

### Quality Control, Data Entry, and Analysis

Data were double entered by different entry clerks and compared for consistency using EpiInfo version 3.3.2 (Centers for Disease Control and Prevention [http://www.cdc.gov/EpiInfo]). Statistical analysis was conducted using Stata version 8.2 (Stata Corporation [http://www.stata.com]). Pearson χ^2^ was used to assess the age and sex distribution of the sample population. Confidence intervals (CIs) for the point estimates were derived using the Huber/White sandwich estimator of variance to adjust for the clustering effects of trachoma at the household level [12]. To derive population estimates of trachoma burden, prevalence of signs of trachoma were adjusted for age and sex according to the population structure. The 95% CIs of the adjusted prevalence estimates were multiplied by the population estimates to derive the lower and upper bounds of those requiring TT surgery. Interobserver agreement of trachoma examiners was assessed using the kappa *(κ)* statistic [13].

### Ethical Considerations

The Sudan People's Liberation Movement Secretariat of Health (SPLM/Health) approved the protocol, and clearance to conduct the surveys was obtained from the local authorities. Verbal consent to participate was sought from the head of the household, and from each individual, and the parents of small children in accordance with the declaration of Helsinki. Personal identifiers were removed from the dataset before analyses were undertaken.

## Results

### Sample Population

Twenty-two villages (clusters) were sampled and 529 households visited. The mean household size was 7.5 (Standard deviation [SD] = 2.5) with household size ranging from three to 16 persons. A total of 3,567 persons were examined (89.7% of those enumerated) of whom 2,017 were children aged less than 15 y and 1,550 were aged 15 y and above. The mean number of persons examined per team per day was 162 (range, 90–228). There were no differences in the age and sex distribution among the 409 persons not examined. Most of the absentees were out at the time of the survey. Of the 3,567 persons included in the analysis, 1,589 (44.5%) were males and 1,978 (55.5%) were females (Table 1). There were more females than males aged 15 y and above among persons examined (Pearson χ^2^ = 36.5; *p* = 0.001). The age range was 2 wk to 80 y, with a mean age of 17.6 y (SD = 17.1) and median age of 11 y (interquartile range, 4–28).

**Table 1:**
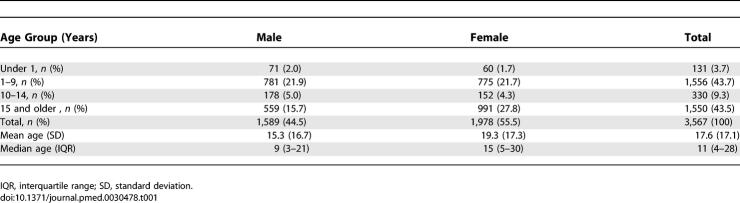
Demographic Characteristics of the Sample Population

### Trachoma Prevalence

**Figure 1:**
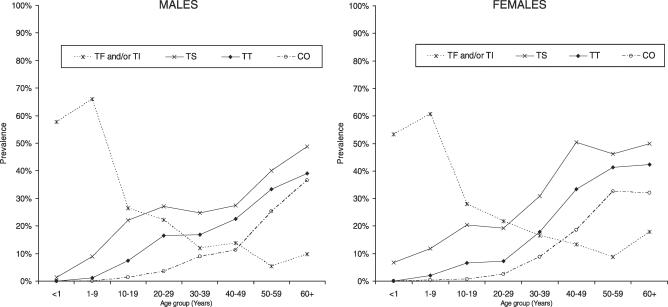
Age and Sex Distribution of Trachoma Signs (All Ages)

**Figure 2:**
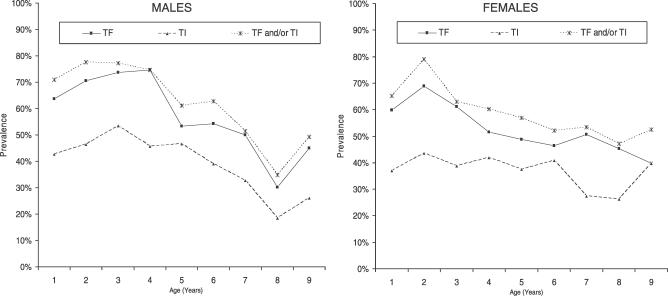
Age and Sex Distribution of Active Trachoma Signs in Children Aged 1–9 y

The prevalence of active trachoma and trichiasis found in this study is shown in Table 2, whereas Figures 1 and 2 show the age- and sex-specific prevalence of trachoma signs. The prevalence of TF in children aged 1–9 y was 57.5% (95% CI, 54.5%–60.4%), whereas prevalence of TI in the same age group was 39.8% (95% CI, 36.3%–43.5%). Active trachoma (TF and/ or TI) prevalence in children aged 1–9 y was 63.3% (95% CI, 60.2%–66.4%). There was no sex difference in active trachoma: odds ratio (OR) = 0.82 (95% CI, 0.66–1.01). The prevalence of active trachoma signs peaked between age 2 and 3 y and then declined (Figures 1 and 2). In persons aged 10 y and above, there was a marked decline in the prevalence of active trachoma signs with prevalence of approximately 13% in persons aged 30 y and above (Figure 1).

**Table 2:**
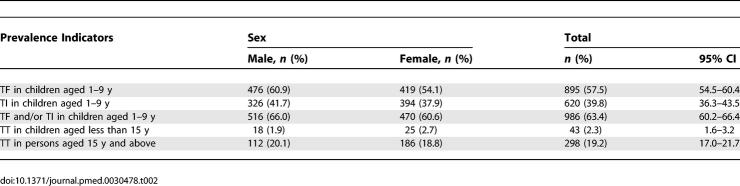
Key Trachoma Prevalence Indicators by Gender

The overall prevalence of TS was 19.8% (95% CI, 18.0%–21.7%). TS was found in 11.5% (95% CI, 9.9%–13.4%) of persons aged less than 15 y, and 30.5% (95% CI, 27.6%–33.5%) of persons aged 15 y and above. TS prevalence increased gradually with age (Figure 1) and there was some evidence that females were more likely to have TS compared to males, although this was not statistically significant: *p* = 0.125; OR = 1.19 (95% CI, 0.95–1.50). The overall prevalence of TT in the sample population was 9.6%; (95% CI, 8.4%–10.9%). The prevalence of TT in children aged less than 15 y was 2.3% (95% CI, 1.6%–3.2%), with trichiasis observed in a child aged 4 y. TT prevalence in persons aged 15 y and above was 19.2% (95% CI, 17.0%–21.7%). There was no sex difference in the prevalence of TT (OR = 1.08; 95% CI, 0.80–1.46). Prevalence of TT increased with age (Figure 1). Prevalence of TF in children aged 1–9 y and TT in persons aged 15 y and above varied between villages, ranging from 14.0% to 62.7% and 1.7% to 23.3%, respectively.

Trachoma-related CO was found in 7.3% (95% CI, 6.5%–8.2%) of the sample population (Table 3). Consistent with the findings for TT, prevalence of CO increased with age (Figure 1). There was no sex difference in the prevalence of CO: OR = 1.17 (95% CI, 0.82–1.67). The prevalence of bilateral CO (CO in both eyes) was 3.1% (95% CI, 2.7%–3.6%). This represents the proportion of the sample population that was visually impaired due to trachoma. Interobserver agreement among examiners who participated in the study compared to our standard (F. Ole-Sempele) ranged from 88% to 98%; overall kappa = 0.64. Kappa for individual trachoma grades was: TF = 0.54, TI = 0.53, TS = 0.50, TT = 0.88, and CO = 0.89.

**Table 3:**
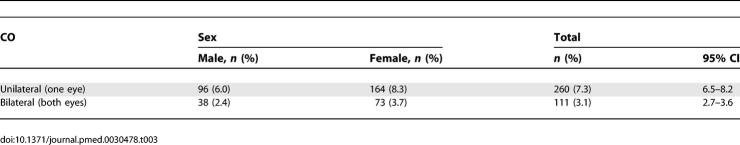
Prevalence of Trachomatous CO (All Ages)

### Trachoma Burden

Estimates of trachoma burden and projected targets for trachoma control interventions are summarized in Table 4. The number of persons with signs of active trachoma in Mankien payam was estimated at 18,417 (lower and upper bounds = 17,354–19,513), whereas the estimated number of persons with TT was 4,557 (lower and upper bounds = 3,946–5,344). CO was estimated to affect 2,799 (lower and upper bounds = 2,268–3,451) persons, of whom 1,354 (lower and upper bounds = 1,038–1,759) had bilateral CO. For planning purposes, the number of persons requiring corrective trichiasis surgery was estimated at 5,344, whereas up to 1,759 persons required rehabilitation for various levels of loss of vision due to trachoma.

**Table 4:**
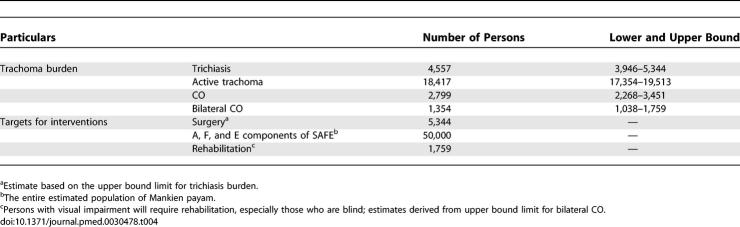
Trachoma Burden Estimates and Program Intervention Targets in Mankien (*n* = 50,000)

## Discussion

This population-based survey provides some of the first reliable data on the prevalence of trachoma in Unity state of southern Sudan, and confirms the initial reports that trachoma is a severe public health problem in Mankien payam. With peace now in place in southern Sudan, these data will be important in establishing health priorities. More than half of children aged 1–9 y had TF (57.5%), whereas TI was present in two fifths (39.8%). The population estimate for prevalence of TT was 9.6% with a fifth (19.2%) of persons aged 15 y and above having this blinding form of trachoma. An exceptionally high prevalence of TT of 2.3% was found in children aged less than 15 y, which suggests intense transmission in early childhood. The overall prevalence of trachoma-related bilateral corneal opacity of 3.1% was alarming. From these data, we estimate that trichiasis surgery is indicated for up to 5,344 persons in Mankien, whereas the entire study population of 50,000 is in need of mass treatment with antibiotics, health education, and environmental improvements to reduce the likelihood of transmission.

We used a two-stage cluster random sampling design to select our sample. This sampling method is practical, efficient, and well suited for populations in which reliable population census data are not available. The random walk method was used to select households. This method may have introduced bias causing overestimation or underestimation of trachoma prevalence at the individual village level, but is likely to have affected the overall prevalence estimates to a lesser extent because these were calculated from 22 villages [14]. Two other methods have been suggested for trachoma assessment: Trachoma Rapid Assessment (TRA) [15] and Assurance Sampling Trachoma Rapid Assessment (ASTRA) [16]. TRA has major limitations: it utilizes a convenience sample, does not allow for estimation of prevalence, and has been found to be inconsistent; and its accuracy is doubtful [17]. ASTRA is based on Lot Quality Assurance Sampling (LQAS) and is recommended for rapid assessment of prevalence of active trachoma. ASTRA has a key advantage of using small sample sizes; however, it is not practical in settings like southern Sudan where up-to-date population census data are not available to generate a stratified random sample. Another drawback is that ASTRA recommends sampling children aged 2–5 y, and thus does not comply with the WHO guidelines of estimating active trachoma prevalence in children aged 1–9 y [18]. In addition, ASTRA does not provide for estimation of prevalence of chronic signs (TS, TT, and CO) and is therefore of limited use in comprehensive assessment of trachoma prevalence.

A response rate of 89.7% was achieved, which was adequate to meet the study objectives and satisfactory, given the logistical and practical constraints of conducting a survey in a postconflict environment. Most of the absentees were aged 15 y and above; therefore, this undersampling of adults is likely to have overestimated the prevalence of TT because absentees were more likely not to have TT compared to persons present. There were no age or sex differences in persons not examined; however, the proportion of males aged 15 y and above was less than of females. This is probably a reflection of the prolonged civil war which has resulted in excess mortality and out-migration of males aged 15–50 y. Mankien boma (12 villages) was excluded from the sampling frame due to insecurity and inaccessibility. Although this is a potential source of bias, we do not expect this to have affected the validity of this survey given the homogenous nature of the payam and the risk factors that predispose these communities to trachoma. Trachoma grading was not supported by nucleic acid amplification techniques, which can detect the presence of C. trachomatis DNA or RNA with great sensitivity and specificity [19]. However, nucleic acid amplification techniques are not routinely used operationally by any program due to the stringent requirements to prevent contamination of samples and the prohibitive cost of processing. Clinical signs are at present the prime basis for program planning and evaluation [20].

The WHO has suggested two key prevalence indicators for determining the public health importance of blinding trachoma: TF prevalence of 10% or more in children aged 1–9 y, and TT prevalence of 1% or more in persons aged 15 y and above [18]. Prevalence of TF and TT revealed in this study exceeds the WHO parameters with TF prevalence being over five times and TT prevalence nearly 20 times. The pattern of signs of trachoma is consistent with findings from other parts of southern Sudan [5,21]. Similar pattern of active trachoma has been observed in Ethiopia, Tanzania, and Mali [22–24]. However, the prevalence of TT in children observed in this study by far exceeds that recorded in other trachoma hyper-endemic settings (e.g., Ethiopia less than 1%, Tanzania = 0.1%) [22,23,25]. The severity of blinding trachoma in this population is further underscored by its early onset and rapid progression. Among 131 infants (age 2 wk to 11 mo) examined, 73 (55.7%) had signs of active trachoma. This early onset coupled with high prevalence of active trachoma in children is consistent with the observed prevalence of scarring. The high prevalence of TS in younger age groups is likely to be as a result of very intense transmission of C. trachomatis and frequent reinfection. This predicts a high prevalence of TT among children and adults with the concomitant risk of blindness early in life; and is well supported by the observed prevalence of TT of 2.3% in children aged less than 15 y. A similar pattern of TT in children has been documented in other parts of southern Sudan [5]. This pattern of trichiasis in children is predictive of a massive future burden of blindness due to trachoma and calls for urgent measures for trachoma control. Therefore, although prevalence of TT in children aged less than 15 y is not a prevalence indicator within the existing WHO guidelines, age-specific prevalence of TT in young children may be a valuable extension of the WHO prevalence indicators. The prevalence of TT in persons aged 15 y and above was 19.2%. Contrary to the usual pattern in which women are two to four times more likely than men to have TT, there was no sex difference observed in this population. This sex equality of suffering from trichiasis may partly be explained by the atypical age–sex structure in this population; however, this phenomenon merits further investigation.

All the communities in Mankien payam are in dire need of trachoma control interventions. Although resources will undoubtedly be inadequate to immediately provide surgery for all, priority might be given to children and those with some residual vision, since that gives the best chance of saving years of useful sight. Mass treatment of communities with azithromycin should be targeted to the entire population of Mankien payam. During the survey, we observed poor facial hygiene in children, many flies on children's faces, and a lack of sanitation and water—all of which are risk factors for trachoma. These observations highlight the need for facial cleanliness and environmental change interventions in these communities. Achieving sustained changes in personal and community hygiene will be a challenge to the implementation of the SAFE strategy, but immediate gains can be made by incorporating hygiene education into the curriculum for the new schools and promoting appropriate disposal of human feces.

In conclusion, Mankien payam has trachoma of severe public health magnitude, and there is an urgent need to implement the full SAFE strategy for trachoma control in this population. The end of the conflict affords a great opportunity for rapid improvements in health infrastructure, access to health care, and implementation of village-based primary health care services in southern Sudan. Although attention will certainly be focused on the “big three” diseases—malaria, HIV/AIDS, and tuberculosis—neglected diseases such as trachoma, Guinea worm [26], and onchocerciasis [27] are all highly prevalent in southern Sudan. It should be possible to integrate control of these neglected diseases into the structure of the new health system and empower this most disadvantaged population to move into the postconflict phase with a greatly reduced burden of preventable diseases.

## Abbreviations

ASTRA: Assurance Sampling Trachoma Rapid Assessment
CI: confidence interval
CO: corneal opacity
GET 2020: Global Elimination of Trachoma by the year 2020
OR: odds ratio
SAFE: surgery, antibiotics, facial cleanliness, and environmental change
SD: standard deviation
TF: trachomatous inflammation-follicular
TI: trachomatous inflammation-intense
TS: trachomatous scarring
TT: trachomatous trichiasis
WHO: World Health Organization
